# How does Pickering Emulsion Pre-treatment Influence the Properties of Wood Flour and its Composites with High-Density Polyethylene?

**DOI:** 10.3390/polym11071115

**Published:** 2019-07-01

**Authors:** Jun Jiang, Changtong Mei, Mingzhu Pan, Jinzhen Cao

**Affiliations:** 1 College of Materials Science and Engineering, Nanjing Forestry University, Nanjing 210037, China; 2 Engineering Research Center of Fast-growing Trees and Agri-fiber Materials of Jiangsu Province, Nanjing 210037, China; 3 MOE Key Laboratory of Wooden Material Science and Application, Beijing Forestry University, Beijing 100083, China

**Keywords:** paraffin Pickering emulsion, wood flour treatment, high-density polyethylene, wood/polymer composites, properties analysis

## Abstract

Silica synergistically stabilized paraffin Pickering emulsion is applied to modify wood flour (WF) for preparing wood/polymer composites. The effect of Pickering emulsion on properties of the WF and its composites with high-density polyethylene (HDPE) is investigated. The impregnation of paraffin Pickering emulsion could significantly improve the WF dispersion in HDPE matrix, resulting in increased melt flow index (MFI). It increased from 1.3 g/10 min (control) to 2.1 g/10 min (Pickering treatment) due to the lubrication of paraffin and rolling friction provided by silica nanoparticles. The hydrophobicity of the WF was improved by the penetration of paraffin and silica in the cell wall, which could consume the hydroxyl groups in WFs via hydrogen bonding. Owing to the well distribution of WFs and silica, the mechanical properties and surface hardness of the composites were enhanced obviously. The optimal tensile strength and impact strength increased 23% (18.28 MPa) and 32% (14.16 KJ/m^2^), respectively. It also could be attributed to the improved interfacial compatibility due to the incorporation of surfactants (Span 80 and Tween 80), which acted as a coupling agent. Furthermore, the silica incorporated in the WF could compensate the negative effect of paraffin on thermal stability of the composites. A model concerning the interactions in the composites was proposed based on the research results.

## 1. Introduction

Natural fibers like wood flour (WF) and nanocellulose are renewable sources and promising reinforcements to replace petroleum-based materials and man-made fibers [1,2,3,4]. Wood flour, as the reinforcement in polymer matrix, has considerable interest in wood/polymer composites (WPCs) fabrication due to its advantages, such as biodegradability, renewability, and low cost [5,6,7]. As a promising alternative for nature wood, WPCs have gained increased recognition in various applications owing to their higher dimensional stability, water resistance, and fungus resistance [8,9]. However, the large amount of hydroxyl groups on the WF makes its surface incompatible with nonpolar polymers, resulting in inferior properties of the composites. In addition, the hydrophilic surface is responsible for WFs aggregation via hydrogen bonding, which causes poor fiber dispersion in the polymer matrix [10]. As a result, the incompatibility and aggregation of WFs produce negative effects on most properties of the composites, such as poor mechanical properties, higher water absorption, and shorter service life. Recently, to overcome these defects, the development of various chemical agents and additives has been applied in WF modification, such as silanes (coupling agent), isocyanates, and organo-montmorillonite [11,12]. However, it is difficult to solve some problems simultaneously and effectively due to the complex system of the composites.

To improve the compatibility between the WF and polymer, most researchers agreed that the surface property of the WF was a significant factor that could affect the fiber-polymer interaction and change the final properties of the composites [13,14]. The coupling agent is a reagent containing both polar and nonpolar groups that acts as a “bridge” to combine the WF and polymer together, resulting in a compatible interface. However, the effect of the coupling agent varies greatly when different polymers are applied [15]. Consequently, the surface modification of WFs is a useful approach to replace or consume the hydroxyl groups on WFs, resulting in better interfacial compatibility between fillers and polymers. These modification techniques include esterification, resin impregnation, WF components extraction, and heat treatment [16,17]. Except for these modifications, surface coating with hydrophobic materials is also a useful method to block inter-bonding between WFs. On the other hand, to further improve the dispersion of WFs in the polymer matrix, appropriate additives are used during WPCs preparation, such as stearic acid, sodium silicate, and mineral oil [18]. However, simultaneously with the positive influence on the WF dispersion, the incorporation of some additives like stearic acid can lead to a decrease in melt flow index (MFI) of the composites [19]. It results in decreased mobility of polymer chains at the interface and shows negative effects on flow behavior of the composites, which is disadvantageous in improving the interfacial compatibility.

Owing to the amphiphilicity, Span and Tween are normal surfactants that can improve the compatibility between hydrophobic and hydrophilic materials [20]. As another candidate, owing to the low cost and hydrophobicity, paraffin is an efficient additive used as a dispersing agent or lubricant in WPCs preparation to reduce agglomeration of fillers in the polymer matrix [21]. Additionally, nanoparticles have unique property in lubrication and tribology, such as anti-wear, reducing friction, and high load capacity [22]. Some investigations showed that the addition of nanoparticles to lubricant oil significantly improved the reducing-friction performance. Li et al. [23] used silane to modify silica nanoparticles and investigated their tribological properties. It showed that modified silica had good dispersion and stability in organic solvents with potential applications as lubrication additives. Peng et al. [24] found that oleic acid modified silica nanoparticles used as liquid paraffin additives had better tribological properties in terms of load-carrying capacity, anti-wear, and friction reduction. Therefore, it is promising to improve the WF dispersion in polymer matrix by addition of nano-silica and paraffin.

The basic idea of the present research is to combine the useful modifiers in the form of Pickering emulsion, which is rarely reported for preparing wood/polymer composites. In our previous study, paraffin Pickering emulsion was successfully prepared and used for solid wood treatment [25]. The silica and paraffin provided a synergistic positive effect on hydrophobicity, surface hardness, and mechanical properties of treated wood. Therefore, the emulsion components could also provide synergistic and positive effects on interfacial compatibility and WFs dispersion in polymer matrix. Pickering emulsion is an emulsion stabilized by solid particles, in which traditional surfactants should be substituted or partially substituted by solid particles. Such kinds of emulsions can be formed in the case of oil-in-water (O/W), water-in-oil (W/O), or multiple emulsions. Compared with emulsions stabilized by surfactants via reducing the O/W interfacial tension, solid particles stabilize the oil droplets by providing a steric barrier at the interface [26]. Silica nanoparticles are widely applied in stabilizing Pickering emulsion. The particles should be partially wetted by both the water and oil phases for an effective emulsification. Due to the hydrophilic surface of the pure silica, the partially hydrophobic silica surface should be obtained by grafting or absorption of non-polar organic groups [27]. For wood or WF modification, silica is an environmentally-friendly modifier with various positive effects [28]. Incorporating silica nanoparticles into polymer-based composites, could improve the flexural strength, impact strength, and surface hardness of WPCs [29].

Herein, hydrophilic silica nanoparticles were used as solid stabilizers for stabilizing paraffin Pickering emulsion. Span and Tween were added as emulsion stabilizers to help the adsorption of silica at oil/water interface. They were also viewed as compatibilizers to improve the compatibility between fillers and polymer matrix. WFs were impregnated by the emulsions with or without silica stabilizers. The effect of Pickering emulsion on the properties of WFs was determined by Fourier-transform infrared (FTIR) spectroscopy, scanning electron microscope (SEM) combined with energy-dispersed X-ray analysis (SEM-EDXA), and the moisture adsorption. Additionally, the melt flow index (MFI) was measured to evaluate the WF dispersion in the polymer matrix. Furthermore, to clarify the influence of paraffin Pickering emulsion on properties the composites, the overall performances were determined by water absorption (WA), thermal stability, mechanical properties and microstructure of impact fracture surface. The main purpose of this study was to characterize the properties of the treated WF and its composites, and to determine whether or not the paraffin Pickering emulsion could enhance the properties of WPCs.

## 2. Materials and Methods

### 2.1. Materials

The WFs of poplar (*Populus tomentosa Carr.*) were collected from wood sawdust with a mesh size of 10–60. They were oven-dried at 103 °C for 48 h to a consistent weight. The HDPE (0.95 g·cm^−3^) was provided by Sinopec Yangzi Petrochemical Co., Ltd, Nanjing, China. The liquid paraffin (purity is higher than 99%), Tween 80 (HLB = 15), and Span 80 (HLB = 4.3) were provided by Beijing Chemical Ltd., Beijing, China. Hydrophilic silica particles (Degussa AG, Frankfurt, Germany) were delivered in powder form with specific surface area 220–300 m^2^·g^−1^ and 20–30 nm particle size.

### 2.2. Pickering Emulsion Preparation and WFs Treatment

**Figure 1:**
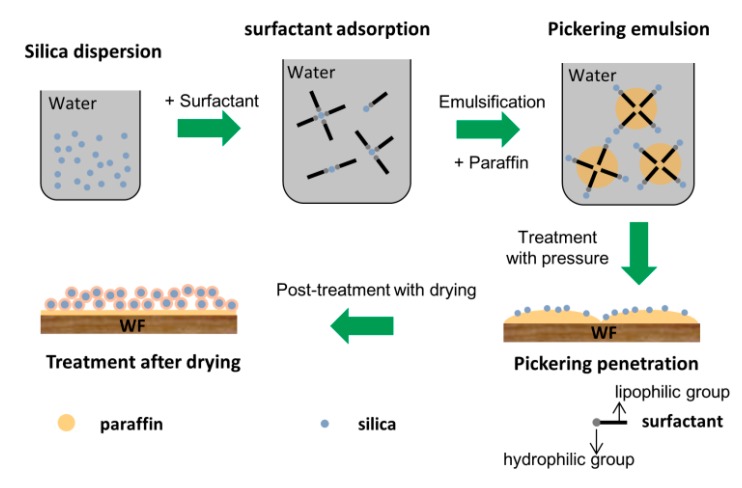
Schematic illustration for Pickering emulsion preparation and wood flour (WF) treatment.

The method applied to prepare paraffin Pickering emulsion can be found in our previous study [25]. Silica dispersion with 0.5 wt % concentration (based on water mass) was prepared at Ph = 3–4 condition adjusted by 0.1 mol·L^−1^ HCl solution. Tween 80 (1.5 wt %) and Span 80 (1.3 wt %) were added into the silica dispersion and gently stirred at a speed of 500 rpm for 1 min. Then, the oil phase (liquid paraffin; 1:5 by vol) was added into the mixture and pre-emulsified at 5000 rpm for 5 min. Afterwards, the pre-emulsion was further treated at 45 MPa for 5 min in the high-pressure homogenizer. WFs were first placed into a beaker in a treating tank and vacuum was applied at 0.01 MPa for 30 min. Then, WFs were completely submerged into Pickering emulsion and pressurized at 0.6 MPa for 40 min. Thereafter, the treated WFs were taken out and dried in an oven at 103 °C to a constant weight (Figure 1). Furthermore, the paraffin emulsion stabilized by only Tween 80 and Span 80 was prepared for WFs treatment. The WFs treated by different emulsion systems were labeled as Pickering emulsion treatment and Paraffin emulsion treatment, respectively.

### 2.3. WF/HDPE Composites Fabrication

The WF/HDPE composites contained 40 wt % of untreated or emulsion treated WFs and 60 wt % HDPE. They were blended in a high-speed mixer (SHR-10a, Huaming Machinery Co., Zhangjiagang, China) at a rotating speed of 3000 rpm for 5 min. The mixture was then dried at 103 °C for 2 h and extruded using a counter-rotating twin-screw extruder (HTY-30, Rubber Machinery Factory Co., Nanjing, China). The corresponding temperature profile along the extruder barrel was 100 °C/115 °C/120 °C/125 °C/125 °C/135 °C/150 °C/150 °C, respectively, and the screw speed was 32 rpm. The extrudate was granulated using a chipper. Thereafter, the granules were injection-molded into standard mechanical test specimens with 175 °C injection temperature and 5 MPa extrusion pressure. The density of the composites was 0.86 g·cm^−3^.

### 2.4. Analytical Methods Applied

The chemical groups of untreated and treated WFs were examined by FTIR analyses (Vertex 70x, Bruker, Billerica, MA, USA). The samples were mixed with KBr in a weight ratio of 1:100 before spectrum collection.

The morphologies of untreated and treated WFs as well as the impact fracture surface of the composites were observed by SEM analysis (S-3400, Hitachi, Tokyo, Japan, 10 kV). The samples were sputter-coated with gold prior to observation. EDXA (7021-H, Horiab, Kyoto, Japan) was performed in mapping mode with an accelerating voltage of 15 kV and 10 nA. The images and distribution of Si element, which was mainly from silica for the Pickering emulsion treated WF, were captured digitally for further analysis.

Prior to the moisture adsorption test, all the WF samples were dried in an oven at 103 °C until they reached a constant weight. Untreated and emulsion treated WFs with 2 ± 0.01 g were placed in a tinfoil box and then kept in desiccators with distilled water at 25 °C for 30 days. The weights of the WFs were recorded periodically, and the moisture adsorption value was calculated to evaluate the hydrophobicity of WFs. For analyzing the WF dispersion in HDPE matrix, melt flow index (MFI) was measured according to ASTM D 1238 with a loading of 2.16 kg at 190 °C. The capillary diameter was 2.08 mm.

The water absorption of WF/HDPE composites were carried out according to the Chinese standard GB/T 17657-2013. Four samples with the size of 50 × 50 × 4 mm^3^ were completely immersed in water at 20 ± 2 °C. The water absorption was calculated based on the weight percent gains after 6, 24, and 48 h, and thereafter at 48 h intervals with removing of excess water on the surface.

Thermogravimetric analysis was conducted to determine the thermal stability of the composites. The samples (appr. 6 mg) were placed in open Pt-crucibles (TG300, Seiko Instruments, Chiba, Japan) and heated from 30 to 600 °C at 10 °C·min^−1^ in N_2_ atmosphere.

The flexural tests were carried out according to the Chinese standard GB/T 9341-2000, which involves a three-point bending test at a speed of 1 mm.min^−1^. The size of the samples was 80 × 10 × 4 mm^3^. Six samples of each group were tested in each run. The modulus of rupture (MOR) and modulus of elasticity (MOE) were calculated to evaluate the flexural properties. The tensile strength (TS) tests were carried out according to the Chinese standard GB/T 1040-1992 at a speed of 2 mm.min^−1^. The size of the samples was 150 × 10 × 4 mm^3^. Six specimens of each group were tested for standard deviations. The impact strength (IS) tests were carried out according to Chinese standard GB/T 16420-1996. Six replicates with the size of 80 × 10 × 4 mm^3^ were tested for each group. Surface hardness (Shore D hardness according to ASTM D2240) was determined on the durometer (TH 210, Beijing TIME High Technology Ltd., Beijing, China). Each sample was measured 10 times.

## 3. Results and Discussion

### 3.1. Microstructure of WFs

**Figure 2:**
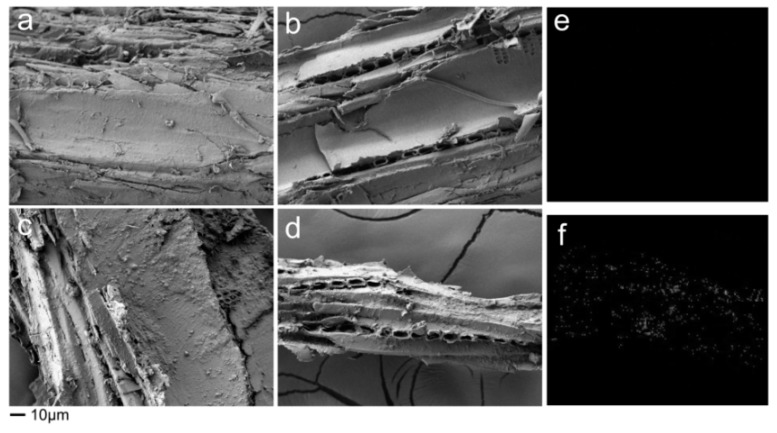
SEM and EDXA images of untreated and emulsion treated WFs. (**a**) without treatment; (**b**) paraffin emulsion treatment; (**c**) and (**d**) Pickering emulsion treatment; (**e**) Si distribution of (**b**); (**f**) Si distribution of (**d**).

The average value of weight percentage gain of WFs after treatment was around 18%, indicating the emulsion could be successfully impregnated into WFs. Figure 2 illustrates the morphologies of untreated and treated WFs. For untreated WFs (Figure 2a), some fiber protrusions were observed. After paraffin emulsion impregnation, all specimens displayed smooth surfaces due to the coverage of paraffin (Figure 2b). After Pickering emulsion treatment (Figure 2c,d), some continuous layers of silica could be found on the surface of cell lumens (Figure 2e,f). This indicated that silica could exist like a continuous film and uniformly covered the WF surface rather than existing as separate particles. This was because the presence of liquid paraffin and surfactants adsorption on the silica surface. These two modifiers could be treated as a medium and compatibilizer, respectively, to improve the silica mobility on the WF surface. On the other hand, some silica particles could fill the pores in WFs (Figure 2d) and consume the –OH groups in WFs with hydrogen bonding, which could improve the hydrophobicity of WFs. This was also detected by others [25,28].

**Figure 3:**
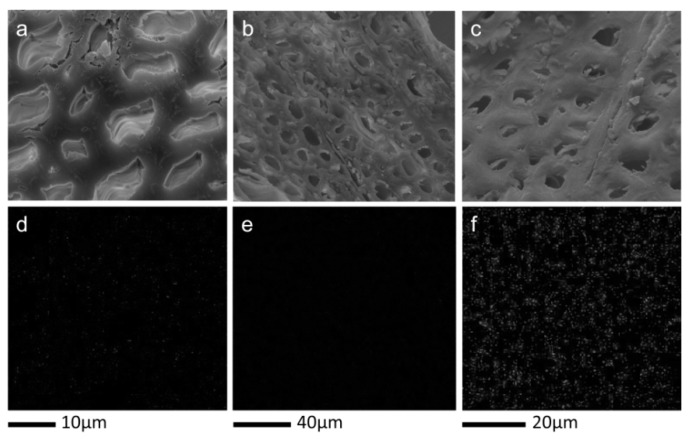
SEM and EDXA images of cross sections of untreated and emulsion treated WFs. (**a**) and (**d**) without treatment; (**b**) and (**e**) paraffin emulsion treatment; (**c**) and (**f**) Pickering emulsion treatment.

The Si distribution images demonstrated whether the silica particles were deposited in the cell wall or not. Compared with untreated and paraffin emulsion treated WFs (Figure 3a,b), the presence of Si was obvious on the cross section of WFs treated by Pickering emulsion (Figure 3c,f). That is, the silica particles were not only in the cell lumens, but also penetrated into the cell wall. It is beneficial for improving properties of the WF as well as its composites. Compared with untreated WFs (Figure 3d), for paraffin emulsion treated WFs, almost no Si distribution could be found (Figure 2e and Figure 3e), indicating the successful coverage of paraffin on the internal surface of WFs.

### 3.2. FTIR Characterization

**Figure 4:**
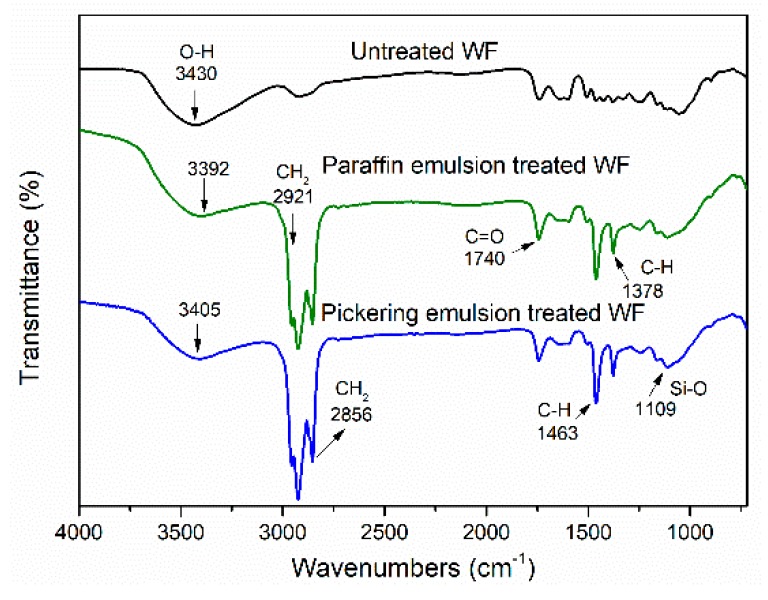
Fourier-transform infrared (FTIR) characterization of untreated and emulsion treated WFs.

The peak at 3430 cm^−1^ for FTIR spectra of untreated WFs (Figure 4) was due to the –OH stretching [30,31]. After treatment, the intensities of these bands decreased because of the hydrophobic paraffin penetration. Furthermore, the broadening band between 3550 and 3200 cm^−1^ was interpretable as hydrogen bonds between the silica and WF [28,32]. Moreover, the -OH bands shifted to lower wavenumbers because of the domination of hydrogen bonds between the WF and surfactants (Span 80 and Tween 80) or silica, respectively [33,34]. The bands appearing at 2921 and 2856 cm^−1^ for emulsion treated WFs were typical for CH_2_ asymmetric stretching and CH_2_ symmetric stretching, respectively, indicating the paraffin penetration [35]. The bands at 1740, 1463, and 1378 cm^−1^ appeared after emulsion treatment, which was due to the C=O stretching vibration and C–H scissoring vibration, respectively. These changes could be attributed to the incorporation of Span 80 and Tween 80 [36]. Probably, there was a combination between the silica and WF via Si–O–Si or Si–O–C covalent bonds. Si–O–Si symmetric stretching vibration (1100–1000 cm^−1^) could overlap with the Si–O–C bonds. The silica sol modified wood also showed these characterizations [37].

### 3.3. Moisture Adsorption of WFs

**Figure 5:**
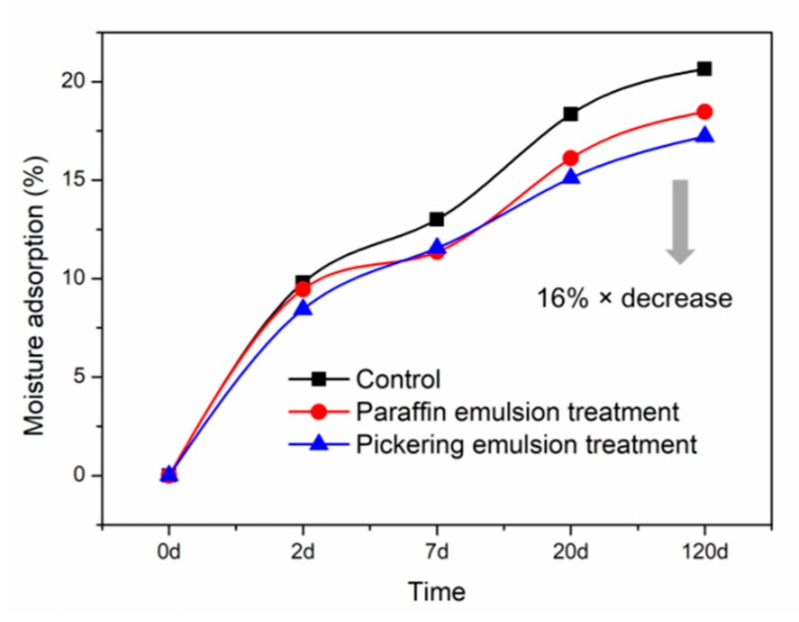
Moisture adsorption of untreated (control) and emulsion treated WFs.

Moisture adsorption was conducted to evaluate the effect of emulsion on hydrophobicity of WFs. The results for untreated and emulsion treated WFs are shown in Figure 5. Compared with the control, the WFs treated with emulsion showed a reduction on moisture adsorption, indicating improved hydrophobicity. Moreover, compared with paraffin emulsion treated WFs, the WFs treated by Pickering emulsion showed optimal hydrophobicity. This was ascribed to the coverage of paraffin and filling effect of silica which could also react with hydroxyl groups on the WF surface to reduce the site for moisture adsorption. Therefore, the Pickering emulsion treated WFs presented the least moisture content. These findings indicated the potential application of paraffin Pickering emulsion in WFs modification for enhancing the hydrophobicity of the resulting WPC.

### 3.4. MFI Analysis

**Figure 6:**
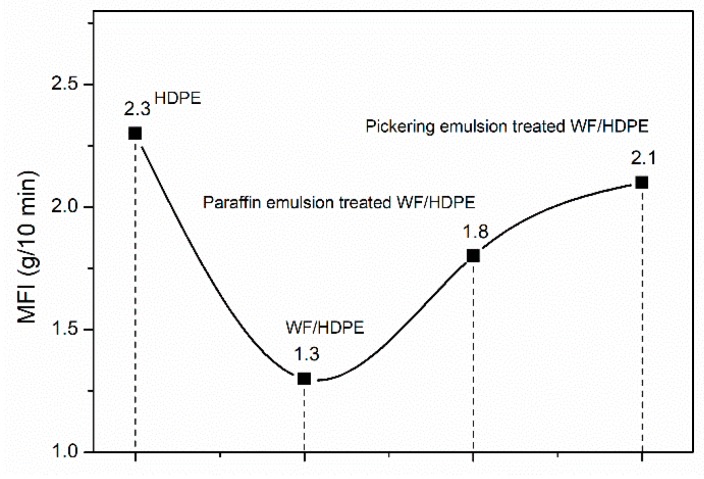
Melt flow index of high-density polyethene (HDPE) and WF/HDPE mixture.

Generally, the higher value of MFI makes polymers flow well around the fillers and it can improve fillers distribution in the polymer matrix, as a result, promoting the interfacial interaction [38]. The MFI values of all samples are shown in Figure 6. Obviously, the MFI value decreased from 2.3 g/10 min (HDPE) to 1.3 g/10 min with the addition of untreated WFs, suggesting the untreated WFs induced inferior mobility. This was because of the rough and hydrophilic surface of WFs (Figure 2a and Figure 5), which might cause WF agglomeration in polymer matrix. However, with the addition of paraffin emulsion treated WFs and Pickering emulsion treated WFs, the MFI value of the mixture increased again. This phenomenon could be explained from two aspects. (1) The paraffin could be viewed as a lubricant, decreasing the friction on the interfaces between WFs and HDPE matrix. (2) The coverage and penetration of paraffin in WFs could improve the hydrophobicity of WFs. It would improve the interfacial compatibility between WFs and HDPE in some extent, resulting in the better dispersion of WFs in HDPE matrix. Both decreased the viscosity of treated WF/HDPE mixture and facilitated the fillers dispersion and mobility in the mixture.

Interestingly, the Pickering emulsion treated WF/HDPE mixture showed the optimal flow behavior. It could be ascribed to the rolling friction between WFs and HDPE matrix, due to the presence of nano-silica film on the WF surface. As seen in Figure 2c, a continuous film uniformly covered the WF surface, which was formed by silica and liquid paraffin. This surface protective film could be viewed as a physical tribofilm that could change the sliding friction to mixing of sliding and rolling friction, resulting in reduced friction and improved mobility of the mixture. Similar phenomenon was found in the study where the nanoparticles were added in lubricant [39].

### 3.5. WA of Composites

**Figure 7:**
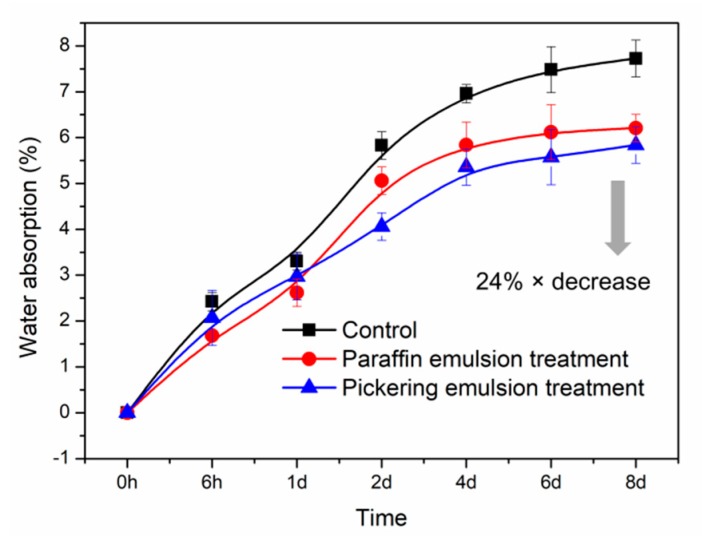
Water absorption of untreated (control) and emulsion treated WF/HDPE composites.

Natural fiber incorporation is mainly responsible for the high water absorption of the polymer-based composites. Compared with the control, the hydrophobicity was improved for the composites reinforced by emulsion treated WFs (Figure 7). The WA decreased clearly from 7.74% (control) to 5.83% (Pickering emulsion treatment). This could be due to the barrier effect of paraffin and the filling effect of silica nanoparticles, inhibiting the water absorption and penetration. Notably, in the range from 0 to 24 h, the WA values of the composites with Pickering pre-treatment were bigger than that of the composites with paraffin emulsion pre-treatment. After that, the value for the former increased slowly. This might be attributed to the exposure of some hydroxyl groups on the silica surface, providing some sites for water-uptake in the initial stage. However, after 24 h immersion, the pore-filling effect of silica dominated the water absorption, namely, it could further prevent the water penetration with the help of paraffin. It also suggested that the better mobility of the liquid paraffin and the nano-sized silica allowed them to diffuse deeply in the WFs, which led to more complete filling of cavities and blocking of hydroxyl groups.

Importantly, the well dispersion of emulsion treated WFs and better interfacial compatibility provided conditions for the improvement on hydrophobicity. It was reported that a well-dispersed mica silicate/poly(e-caprolactone) composite showed reduction in water vapor permeability compared to pure polymers [40]. These results confirmed our previous assumption that paraffin could be used as a dispersing agent to reduce WFs agglomeration and to construct the hydrophobic barrier in the WF, while the silica nanoparticles filled the pores in WFs or interfaces between WFs and polymer matrix to prevent the water penetration.

### 3.6. Mechanical Properties and Surface Hardness

**Figure 8:**
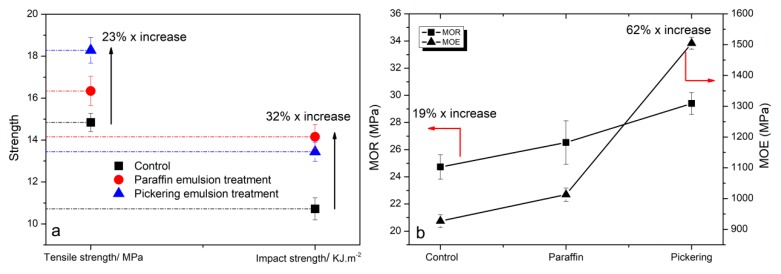
Mechanical properties of untreated (control) and emulsion treated WF/HDPE composites (**a**) tensile strength (TS) and impact strength (IS); (**b**) modulus of rupture (MOR) and modulus of elasticity (MOE).

The mechanical properties of WPCs are illustrated in Figure 8. Compared with the control, significant improvements were obtained after emulsion treatment. The tensile strength (TS) is more sensitive to matrix properties and the interface interaction, while the impact strength (IS) is a balance in properties between the matrix and fillers [41]. After Pickering emulsion treatment, the composites demonstrated a record value in TS (18.28 MPa), which increased by 23% compared with the control (14.84 MPa). However, the optimal IS value (14.16 KJ/m^2^) was obtained from paraffin emulsion modification, which increased by 32% compared with the control (10.72 KJ/m^2^). Impact strength depends largely on the polymer matrix ductility. It is the energy absorption capability during fracture, which represents the interfacial shear strength and bonding of composites. These results could be ascribed to two factors. (1) The liquid paraffin could help the WF to disperse well in polymer matrix. It could avoid WFs aggregation to form local stress concentration during loading, resulting in improved TS and IS. Moreover, paraffin acted as a lubricant to facilitate the polymer ductility. (2) The surfactants could improve the interfacial bonding that facilitated the transferring of impact energy to fillers and consumed the energy via shear friction at the interface. However, the silica nanoparticles have high thermodynamic surface energy and thus become easy to assemble together to reach a stable state [42]. Hence, for Pickering emulsion treatment, the local stress concentration caused by some silica aggregation could also form during loading, and a decrease was found in IS value compared with the one with paraffin emulsion treatment. A similar result was reported in the study, in which more nano-silica addition could induce decreased impact strength [23]. Additionally, the positive effects of paraffin on mechanical properties of wood should be considered, which have been claimed in the literature [43].

The flexural properties of the polymer-based composites are affected by the properties of constituents and the interface interaction. Compared with the control, the modulus of rupture (MOR) and modulus of elasticity (MOE) for composites with Pickering emulsion pre-treatment increased by 19% (29.4 MPa) and 62% (1504 MPa), respectively. Generally, at a high level of WF dispersion, an improvement in strength of the composites could be observed. The penetration of the paraffin and silica in WFs reduced the friction between fillers and the polymer, which was determined by MFI tests, resulting in better mobility. Therefore, the increased WF dispersion contributed to the homogeneity of the composites, suggesting the smooth stress transfer during loading, providing enhanced mechanical properties. Moreover, the incorporation of the silica showed the reinforcement of flexural properties. The surfactants were interpreted as the interfacial coupling agent between the silica and HDPE matrix. It could cause effectively stress transfer from matrix to the stiff silica particles, resulting in improved flexural properties. A similar result was found in the study, in which silane modified mineral fillers were incorporated in WPCs [26,44].

**Figure 9:**
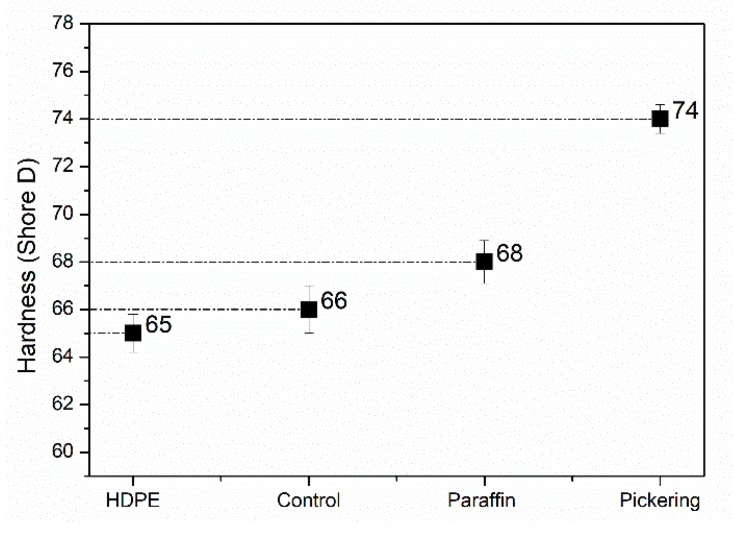
Hardness of HDPE and WPCs made from untreated WFs (control), paraffin emulsion treated WFs (paraffin), and Pickering emulsion treated WFs (Pickering).

The increased hardness of WPCs was due to the improvement in the fillers dispersion and incorporation of silica (Figure 9). Compared with HDPE, the hardness did not change a lot for the control (untreated WF/HDPE), and it increased as paraffin emulsion treatment was conducted. This could be attributed to the improvement in mobility and compatibility of the mixture with the presence of paraffin and surfactants. This phenomenon was also found in the study that polyethylene-co-glycidyl methacrylate (PE-co-GMA) was applied as a compatibilizer in WPC preparation [45]. However, the hardness of the WPC increased again when Pickering emulsion treated WF was used, owing to the incorporation of stiff silica particles. This was also claimed in the study about silica modified wood [25,28].

### 3.7. Microstructure of Impact Fracture Surface

**Figure 10:**
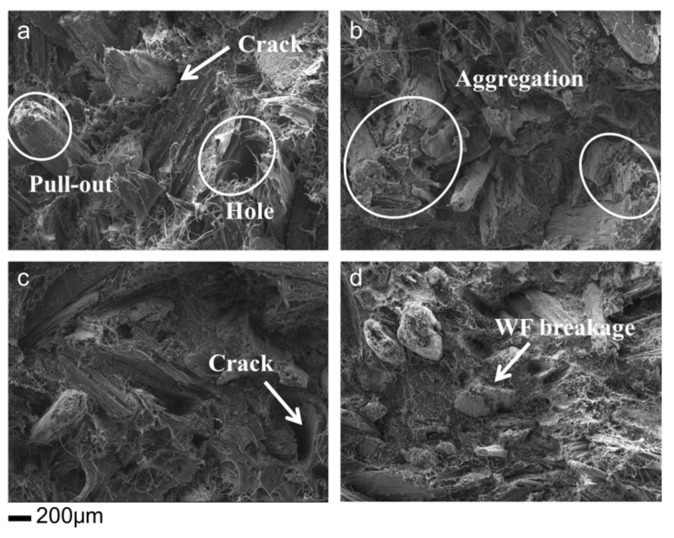
Impact fracture surface of untreated and emulsion treated WF/HDPE composites. (**a**) and (**b**) control: without treatment; (**c**) paraffin emulsion treatment; (**d**) Pickering emulsion treatment.

The interface between fillers and polymer matrix has a significant effect on stress transfer and the spread of cracks in matrix [9]. The microstructures of fracture surface for all composites are illustrated in Figure 10. Analyzing the micrographs provides helpful information about distribution and compatibility of the fillers in composites. As seen in Figure 10a,b, the fiber pull-out was the main failure mode. The interfacial debonding and some holes were also found, which could be ascribed to the weak compatibility. Therefore, it showed a clear gap between the WF and HDPE matrix due to the lack of surface interaction, resulting in easier pull-out of WFs. Moreover, the WF aggregation was observed in polymer matrix owing to the hydrogen bonding. This could cause local stress concentration during the loading, leading to poor mechanical properties (Figure 8).

**Figure 11:**
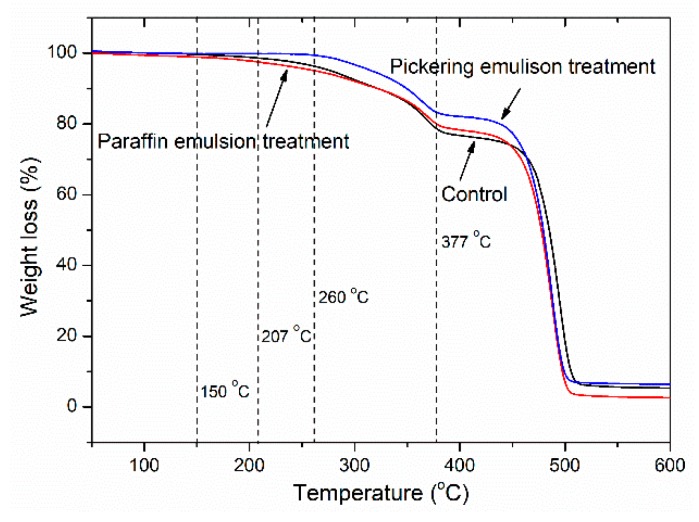
Thermal stability of untreated (control) and emulsion treated WF/HDPE composites.

After emulsion treatment, compared with the control, no filiform protrusions were detected on the fracture surface (Figure 10c,d) due to the penetration of liquid paraffin, which could improve mobility of the WF/HDPE mixture. Moreover, the WF treated by Pickering emulsion showed better dispersion in polymer matrix compared with the control. The result from the MFI provided the evidence for this finding, namely, a higher MFI value made the HDPE flow well to encapsulate the WF and enhance the WF distribution. In addition, the fracture surface of the composites showed integrity without holes and cracks. The WF breakage was observed, indicating the effective stress transfer from matrix to WFs, thus, resulting in increased mechanical strength (Figure 8). Additionally, the WF surface was covered by polymer matrix, suggesting the better interfacial adhesion and improved compatibility. This could be due to the presence of Span 80 and Tween 80, which could work as coupling agents to enhance the interface adhesion [22]. Notably, the roughness of the fracture surface appeared increased in Figure 11d, indicating the dispersion of silica nanoparticles. This phenomenon was also detected in the study, in which the roughness of microstructure increased when silica (5–15 nm) was added in the WPC [45].

### 3.8. Thermal Stability

According to the weight loss (WL) change, the TGA curves are presented in Figure 11. It is hard to absorb water from the atmosphere during the test for polymer-based composites. There was no difference in weight loss for all samples from room temperature up to 150 °C, which might be the removal of absorbed water [46]. An obvious decomposition temperature for the composites with paraffin emulsion pre-treatment was about 150 °C, which could be caused by the decomposition of paraffin [25]. However, for control and the composites with Pickering emulsion pre-treatment, these temperatures were 207 and 260 °C, respectively, indicating the thermal stability could be improved due to the incorporation of silica. After that, the polymer seemed to start decompose at 377 °C.

The residual weights of the samples were 4.2% (control), 1.5% (paraffin), and 5% (Pickering), respectively. It was clear that the paraffin induced the thermal degradation of the composites, while this negative effect could be compensated by the positive effect of silica due to its better thermal stability. Therefore, the composites with Pickering emulsion pre-treatment showed the lowest WL compared with others. Moreover, Span 80 and Tween 80 could play a significant role in improving the interfacial adhesion between fillers and polymer matrix, which delayed the thermal degradation of the composites. The dispersed silica film on the WFs surface (Figure 2c,d) produced difficulty in heat conduction and acted as a mass transport barrier during the decomposition [47].

### 3.9. Model for Composites Formation

**Figure 12:**
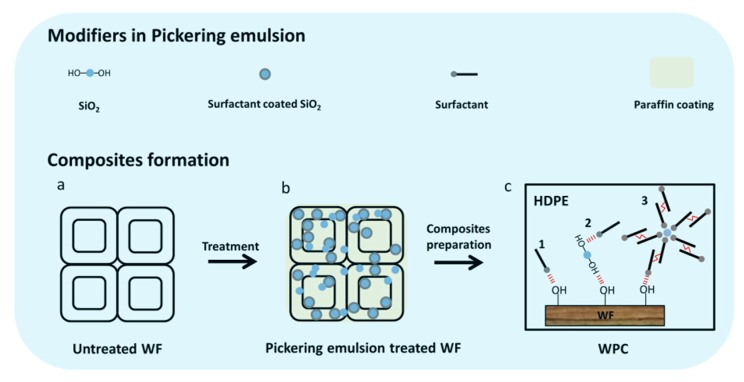
Schematic illustration for interactions in the composites treated by Pickering emulsion.

Silica and liquid paraffin could be distributed in cell lumens and cell walls, resulting in improved properties of the WF and its composites. After treatment, the silica could form a continuous film on the WF surface with the “adhesion” of liquid paraffin, and fill some pores in the WF (Figure 12a,b). Three types of interaction could be determined between the WF and modifiers in the Pickering (Figure 12c) to improve the compatibility between fillers and polymers. (1) Some surfactants (Tween 80) could be adsorbed on the WF surface via hydrogen bonding due to the presence of hydrophilic group (Figure 12c-1). (2) The partially coated silica could react with the WF via the hydrogen bonding between hydroxyl groups, while the lipophilic group of Tween 80 could enhance the compatibility between fillers and HDPE matrix (Figure 12c-2). The hydrophilic-lipophilic balance (HLB) value of Span 80 is 4.3, indicating it prefers to dissolve in oil phase during the emulsion preparation. Therefore, when liquid paraffin was added into the water phase, Span 80 could form reverse micelles in the oil phase. (3) Thereafter, some hydrophilic silica could be adsorbed in the polar core of the reverse micelles by acid-base interaction with the surface sites [48,49]. It can be imagined that within an appropriate range for Span 80 addition, the increase of Span 80 could strengthen adsorbed layer of the reverse micelles due to the interaction between adjacent surfactants (Figure 12c-3), resulting in better steric barrier to improve the dispersion of silica or treated WFs in HDPE matrix. Based on these interactions and the lubricant effect of paraffin, the dispersion of fillers and the interfacial compatibility in the composites could be improved accordingly.

## 4. Conclusions

The treatment of WFs with silica synergistically stabilized paraffin Pickering emulsion and its application in polymer-based composites were successfully conducted. The incorporation of paraffin acted as a lubricant and could result in greater dispersion of WFs in HDPE matrix, leading to reduced aggregation of WFs and improved interactions between fillers and polymer matrix. By changing the sliding frication to rolling frication, the silica nanoparticles played an important role in improving the mobility of WF/HDPE mixture. The adsorption of surfactants (Span 80 and Tween 80) on the silica or the WF surface could improve its dispersion in polymer matrix and interface compatibility between fillers and HDPE, which provided positive effects on the improvement of mechanical properties, surface hardness, and thermal stability. The synergistically stabilized paraffin Pickering emulsion brought benefits from the hydrophobicity of paraffin, the nano-effect of silica particles, and the coupling effect of surfactants. Pre-treatment with paraffin Pickering emulsion shows the potential application in producing functional WPCs with a one-step method. Further developments should be explored, focusing on the effect of silica content on Pickering emulsion properties and its application in WPCs fabrication.
